# Molecular Marker Differences Relate to Developmental Position and Subsets of Mesodiencephalic Dopaminergic Neurons

**DOI:** 10.1371/journal.pone.0076037

**Published:** 2013-10-07

**Authors:** Simone M. Smits, Lars von Oerthel, Elisa J. Hoekstra, J. Peter H Burbach, Marten P. Smidt

**Affiliations:** 1 Molecular Neuroscience, Swammerdam Institute for Life Sciences, University of Amsterdam, Amsterdam, The Netherlands; 2 Department of Neuroscience and Pharmacology, Rudolf Magnus Institute of Neuroscience, University Medical Center Utrecht, Utrecht, The Netherlands; Rutgers University, United States of America

## Abstract

The development of mesodiencephalic dopaminergic (mdDA) neurons located in the substantia nigra compacta (SNc) and ventral tegmental area (VTA) follow a number of stages marked by distinct events. After preparation of the region by signals that provide induction and patterning, several transcription factors have been identified, which are involved in specifying the neuronal fate of these cells. The specific vulnerability of SNc neurons is thought to root in these specific developmental programs. The present study examines the positions of young postmitotic mdDA neurons to relate developmental position to mdDA subset specific markers. MdDA neurons were mapped relative to the neuromeric domains (prosomeres 1-3 (P1-3), midbrain, and hindbrain) as well as the longitudinal subdivisions (floor plate, basal plate, alar plate), as proposed by the prosomeric model. We found that postmitotic mdDA neurons are located mainly in the floorplate domain and very few in slightly more lateral domains. Moreover, mdDA neurons are present along a large proportion of the anterior/posterior axis extending from the midbrain to P3 in the diencephalon. The specific positions relate to some extent to the presence of specific subset markers as Ahd2. In the adult stage more of such subsets specific expressed genes are present and may represent a molecular map defining molecularly distinct groups of mdDA neurons.

## Introduction

Parkinson’s disease (PD) is a common progressive neurodegenerative movement disorder attributed to selective loss of pigmented mesodiencephalic dopaminergic (mdDA) neurons in the substantia nigra compacta (SNc), whereas adjacent mdDA neurons in the ventral tegmental area (VTA) are largely spared [1]. A similar susceptibility to degeneration of SNc DA neurons exists in animal models of PD, indicating a conserved phenomenon across species (for review, see [2]). Although mdDA neurons within the SNc and VTA share their neurotransmitter phenotype, it is now evident that several DA subpopulations can be identified based on anatomy, connectivity, pharmacology, electrophysiological properties and gene expression [3]–[12]. Analysis of a Pitx3-deficient mouse called aphakia (*ak*) has provided evidence that DA neurons react differentially to the ablation of Pitx3 during development [7], [13], [14], indicating that specific vulnerability of mdDA subsets exist already during development. Recently, we have proposed a novel mechanism in which aldehyde dehydrogenase 2 (Ahd2)-mediated RA production is involved in the neuronal development and maintenance of mdDA neurons in the SNc and VTA, providing new insights into subset-specific vulnerability in PD [7], [14].

The generation of cellular diversity in the CNS is initiated by subdividing the neural plate along the anteriorposterior (A/P) axis into regions (fore-, mid-, hindbrain and spinal cord) and along the dorsovental (D/V) axis into longitudinal zones (floor, basal, alar and roof plate). Whereas the midbrain region is recently subdivided, in newest anatomical atlases, in mesomere 1 and 2. The earliest event involved in the commitment and position of SNc and VTA DA neurons along the A/P axis is the establishment of the mid/hindbrain border (MHB) by the Otx2-Gbx2 interface, which sets the caudal limit for the DA neurons [15]–[17] Next, additional transcription factors, including Nurr1, Pitx3, En1/2, Lmx1A and B are required to specify the DA system (for a review, see [18]) In contrast to the caudal boundary, relatively little is known about the rostral boundary of the DA domain [19].

Identity and position of DA progenitor cells in the ventricular zone along the D/V axis is instructed, amongst other signals, by Shh [20], [21]. When DA progenitor cells stop proliferating they become post-mitotic, enter into a differentiation program and start to migrate ventrally along radial glia (eventually with ulterior tangential displacement) to their final destinations in the tegmental mantle [22]–[24]. TH-immunoreactive neurons, which later form the neuranatomical regions, SNc and VTA, are located in both the floor plate and the adjacent basal plate in human and mouse embryos [25]. Thus, DA neurons of the SNc and VTA are positioned in the brain across several regions within their floor and basal plate domain. Differences in the developmental programs of the segmental/longitudinal subdivisions may underlie the specific hallmarks of DA subsets, as reflected by the selective vulnerability of SNc neurons as exemplified in Pitx3 deficiency [13]. In this study, we have determined the segmental and longitudinal positions of mdDA populations in mice in relation to gene expression along the anterior/posterior domain. The position of developing mdDA neurons suggest that specific molecular programs may influence the terminal differentiation of these neurons. We show here that these specific developmental positions link to the specific molecular makeup of mesodiencephalic dopaminergic neurons in the adult state, providing a molecular map of mdDA neuronal subsets.

## Results

### SNc/VTA DA Neurons are Organized in a Continuum Along the A/P Axis Extending from the Midbrain to P3

**Figure 1:**
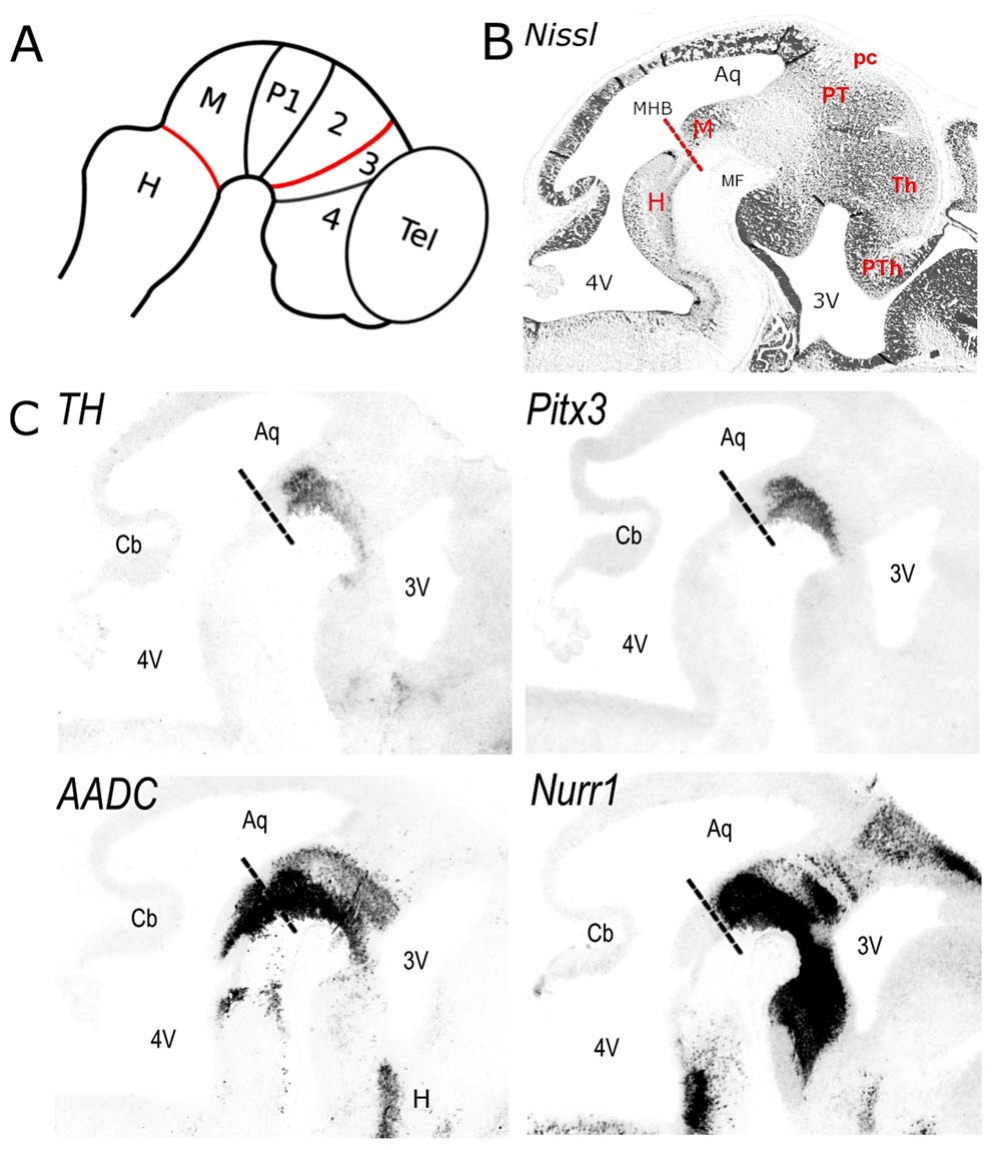
(A) Schematic sagittal representation of the prosomeric model as proposed by Puelles and Rubenstein (2003). The transverse lines represent interneuromeric boundaries in which the mid/hindbrain border (MHB) and the zona limitans intrathalamica, a boundary between the thalamus (P2) and prethalamus (P3) are depicted in red. (B) Nissl stained E13.5 sagittal section for the anatomical orientation. (C) In situ hybridisation for Th, Pitx3, Aadc and Nurr1 on E13.5 sagittal sections. Abbreviations: 2, prosomere 2; 3, prosomere 3; 4, prosomere 4; 3V, third ventricle; 4V, fourth ventricle; Aq, aqueduct; Cb, cerebellum; H, hindbrain; M, midbrain; MHB, mid/hindbrain border (indicated by dashed lines); MF, mesencephalic flexure; P1, prosomere 1; pc, posterior commissure; PT, pretectum; PTh, prethalamus; Tel, telencephalon; Th, thalamus.

To indicate the structural and anatomical organization of the mdDA system along the A/P axis, relative to the neuromeric domains (prosomeres 1-3 (P1-3), midbrain, and hindbrain) as proposed by the prosomeric model (Fig. 1A; [26]) E13.5 sagittal sections stained with different DA markers were analyzed to match the mdDA domain with the ventral position of these neuromeric boundaries. First, Nissl staining was performed for the structural detail of the region examined (Fig. 1B). The following anatomical landmarks used to locate the relevant transverse interneuromeric boundaries were observed: the mid/hindbrain border (MHB), posterior commissure (midbrain/P1 limit), pretectum (P1), the thalamus (previously known as the dorsal thalamus (P2)), and the prethalamus [27] (previously known as the ventral thalamus (P3)). Fully differentiated DA neurons were identified by the expression of Th and Pitx3 (Fig. 1C). Based on the anatomical landmarks identified by Nissl stainings, Th and Pitx3-positive DA neurons were positioned in the most ventral part of the mesencephalic flexure across several neuromeres, including the midbrain, P1, P2 and P3 (Fig. 1C). Interestingly, Aadc expression is induced two days earlier than Th [28] and is suggested to identify younger stage migrating mdDA neurons located more dorsally within the mdDA developmental domain [13]. To determine in which neuromeric domains these developing mdDA neurons were positioned, Aadc expression was analyzed on adjacent sections (Fig. 1C). Confirming the data found for Th, Aadc expression was observed along the anterior/posterior axes, in the midbrain up until P3 in the diencephalon. In addition, expression was observed in the anterior hindbrain and anterior hypothalamus coding in this region the noradrenergic and serotonergic developing neurons. The nuclear hormone receptor Nurr1, which is involved in the molecular specification of DA neurons and is involved in activating Aadc [8], is expressed when proliferating mdDA progenitor cells become postmitotic, and therefore identifies both young-stage and fully differentiated mdDA neurons. Nurr1 expression was observed across the mesencephalic flexure (MF) in the midbrain, P1, P2 and P3, extending in the posterior hypothalamus (Fig. 1C), showing that Nurr1 is not exclusively present in mdDA neurons as earlier suggested [8], [29].

**Figure 2:**
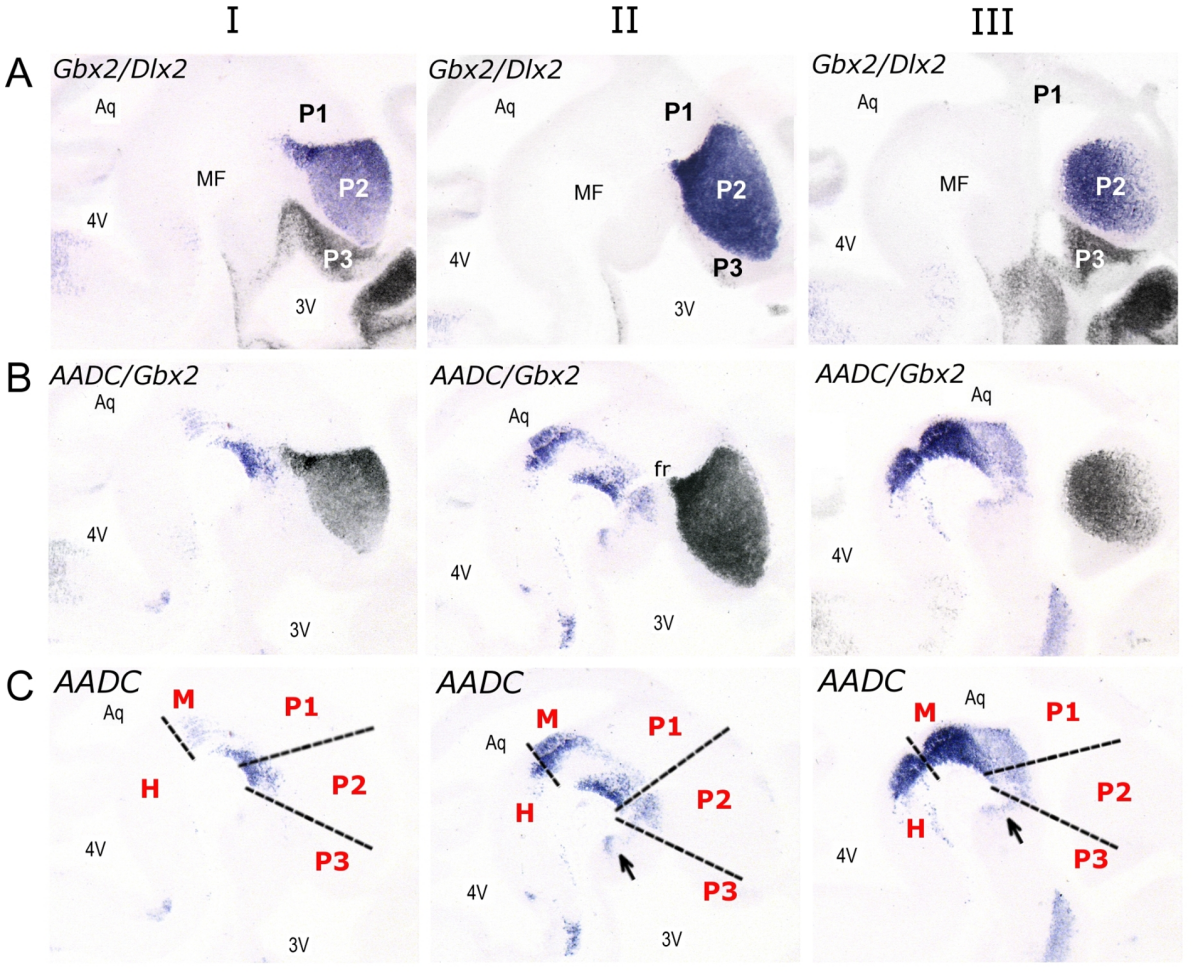
Identification of the transverse interneuromeric boundaries by region specific markers Gbx2 and Dlx2 on E13.5 sagittal sections at three different levels, from lateral to medial, I to III respectively. (A) Overlays of mRNA expression patterns of Gbx2 (purple) and Dlx2 (black) on adjacent sections. Gbx2 is expressed in prosomere 2 (P2) creating a sharp boundary with the Dlx2 expression domain in P3. (B) Overlays of mRNA expression patterns of Aadc (purple) and Gbx2 (black) on adjacent sections. (C) Overlays of mRNA expression patterns of Aadc (purple) and Dlx2 (black) on adjacent sections. (D) The major transverse interneuromeric boundaries are illustrated by dashed lines in E13.5 sagittal sections. Note that Aadc positive neurons are located across multiple segments (M, P1, P2 and P3). Abbreviations: 3V, third ventricle; 4V, fourth ventricle; Aq, aqueduct; H, hindbrain; M, midbrain; MF, mesencephalic flexure; P1, prosomere 1; P2, prosomere 2; P3, prosomere 3.

The position of mdDA neurons in relation to the transverse interneuromeric boundaries, within the mdDA regions of midbrain to diencephalic P3, was analyzed in more detail by comparing Aadc expression to expression patterns of region specific markers on adjacent E13.5 sagittal sections(Fig. 2). The homeobox genes Gbx2 and Dlx2 define transverse segmental boundaries in the forebrain [30]. Gbx2 is expressed in the thalamus (P2) creating a sharp boundary with the adjacent Dlx2 expressing domain in the prethalamus (P3) at the position of the zona limitans intrathalamica, a boundary-cell population that develops between the thalamus and prethalamus (Fig. 2A). Thus, the boundaries of the Gbx2 expressing domain mark the P1 (posterior to Gbx2), P2 (Gbx2 positive) and P3 (anterior to Gbx2) territories (Fig. 2A). Overlays of adjacent sections containing mRNA expression of Aadc and Gbx2 show that Aadc-positive mdDA neurons are located in P1 and P2 at all three levels, and in P3 only at level II and III (Fig. 2B). Taken together, mdDA neurons, as characterized by Aadc, Nurr1 and Th expression in this region, are organized in a continuum extending from the midbrain to P3 (Fig. 2C).

### mdDA Neurons are Mainly Present in the Floorplate and Few in Basalplate Positions

**Figure 3:**
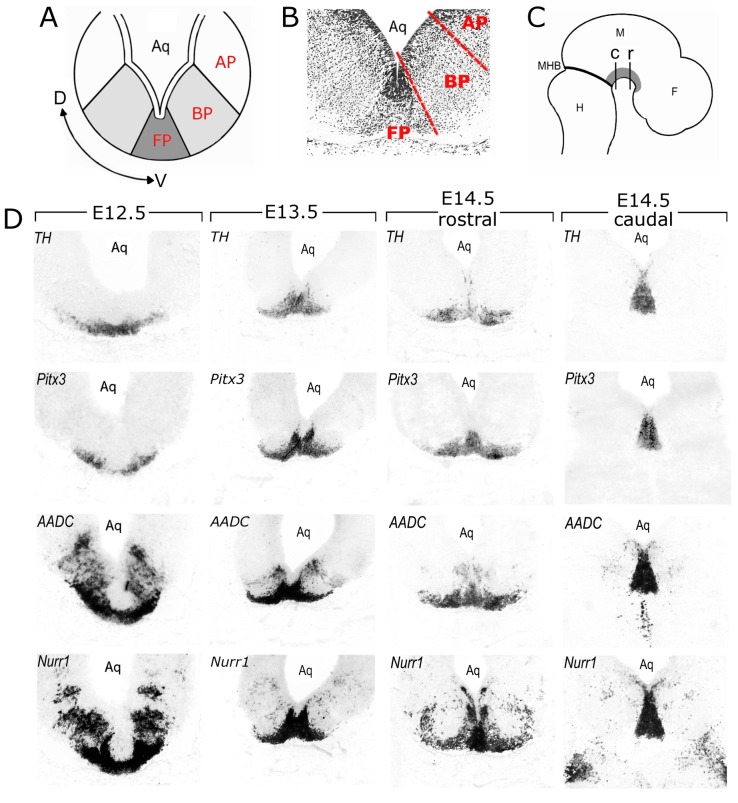
Presentation dorsal-ventral boundaries at the rostral and caudal mesodiencephalon during development. (A) Schematic coronal representation of the dorso-ventral organization of the floor plate (FP), basal plate (BP) and alar plate (AP). (B) Nissl stained E13.5 coronal section for the anatomical orientation. The floor-basal and basal-alar boundaries are shown in red dashed lines. (C) Schematic representation of a sagittal section showing the position of the coronal sections shown in G and F. The position of DA neurons are depicted in grey. (D–G) In situ hybridisation for Th, Pitx3, Aadc and Nurr1 on E12.5 (D), E13.5 (E), anterior E14.5 (F) and posterior E14.5 coronal sections (G). Abbreviations: Aq, aqueduct; F, forebrain; H, hindbrain; M, midbrain; MHB, mid/hindbrain border.

To determine the structural and anatomical organization of the mdDA system along the D/V axis, relative to the longitudinal subdivisions (floor plate, basal plate and alar plate; Fig. 3A–C), coronal sections from E12.5, E13.5 and E14.5 old embryos were stained with different DA markers (Fig. 3 D). First, Nissl staining was performed on E13.5 coronal sections for the structural detail of the region examined (Fig. 3B). The floor plate could be discriminated from the basal plate by the more intense staining in the floor plate. The basal plate could be delimited from the alar plate by the thickness of the ventricular zone, which is distinctly thicker in the alar plate, as previously shown for human embryos [31]. Based on these anatomical boundaries, the mRNA expression patterns of Th and Pitx3 showed that fully differentiated DA neurons are positioned in both the floor and basal plate at all three embryonic stages analyzed (Fig. 3D). Interestingly, Th and Pitx3-positive neurons were restricted to the floor plate in the most caudal part, whereas in the more rostral part these neurons were also present in the basal plate. To determine in which longitudinal zones young mdDA neurons are present within the mdDA specific anterior/posterior domain, Aadc and Nurr1 mRNA expression was analyzed on adjacent sections at E12.5, E13.5 and E14.5 (Fig. 3D). At E12.5, Nurr1 and Aadc were highly expressed in the floor and basal plate immediately below the ventricular zone. At E13.5, Nurr1 and Aadc were highly expressed in the fully differentiated DA neurons (compared with Th and Pitx3; Fig. 3D), but the expression in the dorsal basal plate was decreased. At E14.5, the mdDA area had expanded and the DA neurons could be distinguished based on their anatomical positions. Therefore, we discriminated between rostral and caudal mdDA neurons (Fig. 3D). Nurr1 and Aadc were still highly expressed in the fully differentiated DA neurons, but Aadc expression was nearly absent in the basal plate immediately dorsal to the fully differentiated DA neurons, except for some cells in the rostro-medial part and few cells in the caudal midbrain. Expression of Nurr1 was observed in the most dorsal and most ventral part of the basal plate at the rostral level and few Nurr1-positive cells were observed in the basal plate at the caudal level.

**Figure 4:**
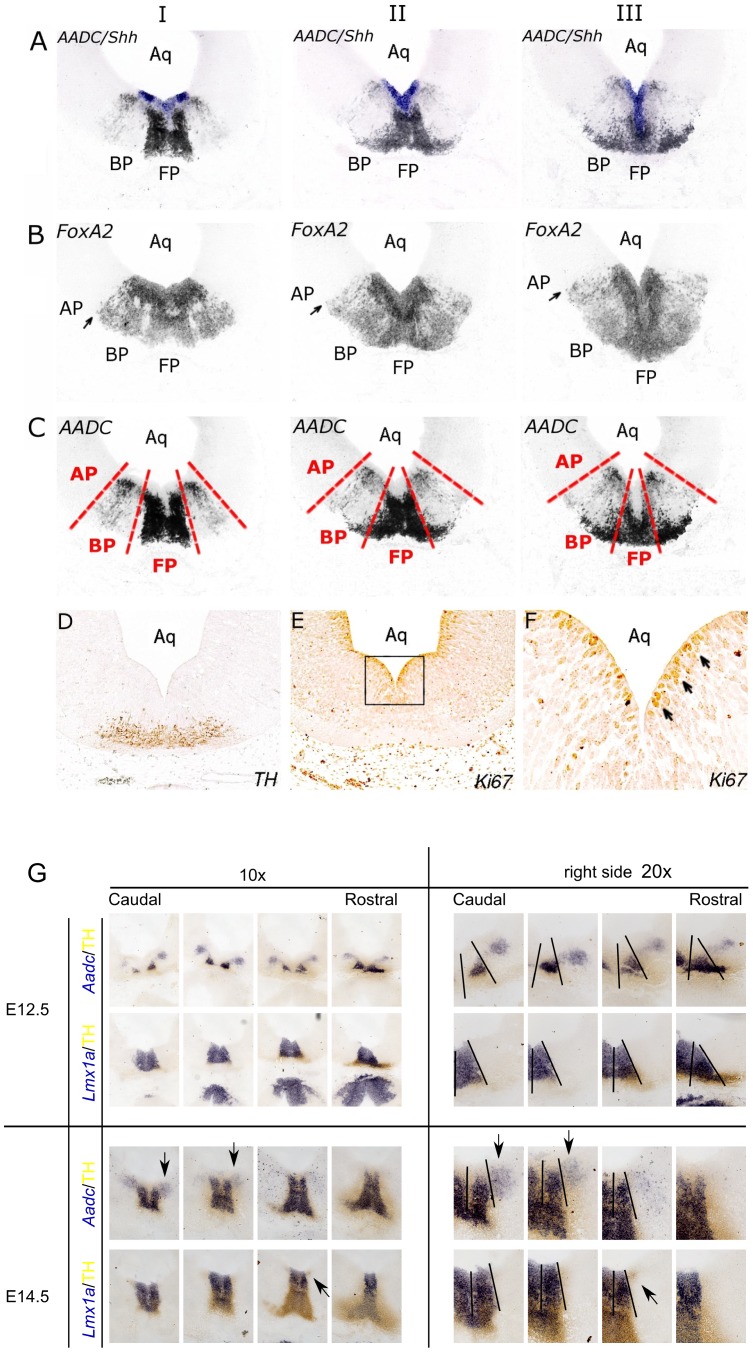
Identification of the longitudinal subdivisions by region specific markers Shh and FoxA2 on E13.5 sagital sections at three different levels, from posterior to anterior, I to III respectively. (A) Overlays of mRNA expression patterns of Shh (purple) and Aadc (black) on adjacent sections. Shh is highly expressed in the ventricular zone of the basal plate, whereas the signal in the floor plate is less intense. (B) In situ hybridization for FoxA2 on adjacent sections marking the basal-alar plate boundary, as indicated arrows. (C) The major longitudinal subdivisions are illustrated by dashed red lines in E13.5 sagittal sections, in which in situ hybridization for Aadc identifies the putative DA neurons. Note that Aadc positive neurons are located in the floor and basal plate. (D) TH imunostaining on an E12.5 coronal section for the identification of fully differentiated mdDA neurons. (E) Ki67 immunostaining on adjacent E12.5 coronal sections identifies the proliferative cells in the ventricular zone. (F) Higher magnification of the boxed area in panel E. Note that the proliferating cells in the ventricular zone are orientated radially (see arrows). (G) Co-localization of the floorplate marker Lmx1a with Aadc and Th at E12.5 and E14.5. Note the expression of TH and Aadc outside the Lmx1a expression domain (arrows) Abbreviations: AP, alar plate; Aq, aqueduct; BP, basal plate; FP, floor plate.

The position of young mdDA neurons in relation to the longitudinal subdivisions was analyzed in more detail by comparing Aadc expression to expression patterns of region specific markers Shh, FoxA2 (HNF3-β) on adjacent E13.5 coronal sections at three different levels, from posterior to anterior, I to III respectively (Fig. 4A–D). Shh is expressed at very early stages in the ventricular zone of the floor plate and later in the midbrain and P1-3 basal plate, whilst the signal in the floor plate diminishes. This transition of Shh expression coincides with the boundary between floor and basal plate [32], [33]. Overlays of adjacent sections containing mRNA expression of Aadc and Shh showed that Aadc positive neurons are located in the floor and basal plate at all three levels just below the ventricular zone (Fig. 4A). To determine whether the slightly more dorsal expression of Aadc, as compared to Shh, is basal plate or alar plate, FoxA2 expression was studied on adjacent sections. FoxA2 is expressed in an adjacent domain to alar plate marker Otx1, thereby marking the alar-basal plate boundary (Fig. 4B). Overlays of adjacent sections containing mRNA expression of Aadc and FoxA2 showed that Aadc was not expressed in the alar plate (data not shown). Thus, these data indicate that Aadc positive neurons are located in both the floor and basal plate along the A/P domain (Fig. 4C), and suggest that young mdDA neurons are present in these domains as was shown for the fully differentiated neurons. Proliferative neurons in the ventricular zone of coronal E12.5 sections were identified by Ki67 immunoreactivity (Fig. 4E and F). These proliferating cells were orientated radially, suggesting that Aadc positive neurons just below the ventricular zone in the basal plate might be generated in the ventricular zone of the basal plate. Finally, in order to independently identify mdDA neurons in the basal plate domain (outside the Lmx1a domain) we performed double ISH-IMHC experiments with Lmx1a, Aadc and TH (Fig. 4G). The data confirmed our previous observations. Aadc and TH are present outside the Lmx1a expression domain at the most dorsal position of the ventral mesencephalon. Taken together, early as well as fully differentiated mdDA neurons are mainly present in the floorplate domain and few can be identified in the basal plate domain as identified by Aadc and Th.

### Precursor Cells Aligning the Third Ventricle are in Proximity to Aadc Positive Neurons Located in the Posterior Diencephalon

**Figure 5:**
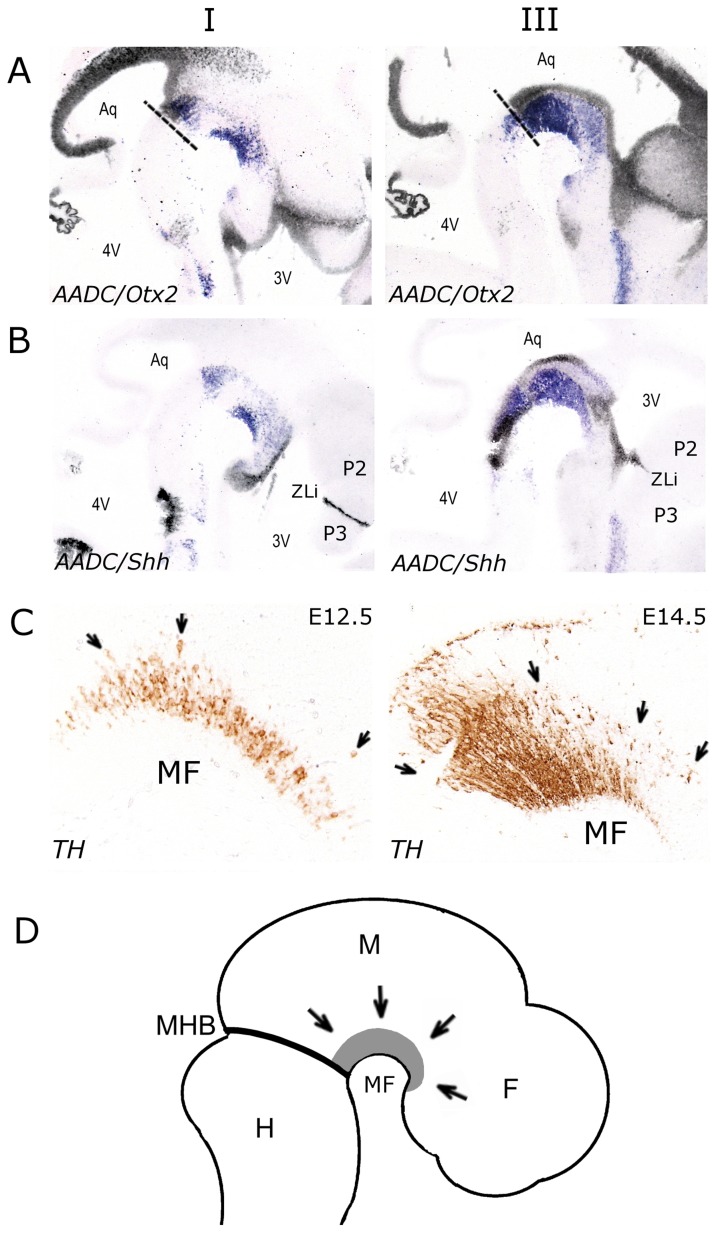
Analysis of ventricular zone markers Otx2 (A) and Shh (B) in E13.5 sagittal sections at two different levels, lateral (I) and medial (III). (A) Overlays of mRNA expression patterns of Otx2 (black) and Aadc (purple) on adjacent sections. (B) Overlays of mRNA expression patterns of Shh (black) and Aadc (purple) on adjacent sections. Note that young Aadc positive neurons in the posterior diencephalon are in proximity with the ventricular zone aligning the third ventricle. (C) High magnification of the mesencephalic flexure (MF) in sagittal E12.5 and E14.5 sections immunostained with TH. Anterior is to the right and posterior to the left. Note that TH-immunoreactive neurons are radially orientated along the A/P axis (see arrows). (D) Schematic representation of a sagittal E13.5 embryo, in which mdDA neurons are depicted in gray. The arrows indicate the radial migration of DA neurons. Abbreviations: 3V, third ventricle; 4V, fourth ventricle; Aq, aqueduct; F, forebrain; H, hindbrain; M, midbrain; MF, mesencephalic flexure; MHB, mid/hindbrain border; P1, prosomere 1; P2, prosomere 2; P3, prosomere 3; ZLi, zona limitans intrathalamica.

During early development of the CNS, immature neurons which completed their final mitosis in the ventricular zone migrate to their final destination. Fully differentiated mdDA neurons located across the MF are thought to be generated by proliferative cells in the ventricular zone aligning the midbrain cavity, the cerebral aqueduct (aqueduct of Sylvius), a canal that communicates between the third and fourth ventricles. The cavity of the diencephalon forms the greater part of the third ventricle, communicating posteriorly with the aqueduct. Since SNc/VTA DA neurons are located across the A/P axis in the midbrain and posterior diencephalon, a subset of these neurons might also be present below the ventricular zone aligning the third ventricle. To identify the ventricular zones, which are in proximity to Aadc positive neurons, sagittal E13.5 sections were stained with ventricular zone markers implicated in the neurogenesis and progenitor identity of cells in the midbrain, Otx2 and Shh [34], [35]. Otx2 is expressed in every dorsal and most ventral regions of telencephalon, diencephalon and mesencephalon with a sharp limit at the MHB (Fig. 5A). Overlays of adjacent sections containing mRNA expression of Aadc and Otx2 indicate that Otx2-positive cells in the ventricular zone aligning the third ventricle are in proximity to Aadc positive neurons in the posterior diencephalon (Fig. 5A,I). As expected, Otx2 expression in the ventricular zone aligning the aqueduct was in proximity of Aadc positive neurons in the midbrain (Fig. 5A, III). Shh is expressed in the ventricular zone along the entire A/P axis (hindbrain, midbrain, diencephalon (P1-3)) with a prominent dorsal extension at the zona limitans intrathalamica, which lies at the boundary between P2 and P3 ([32]; Fig. 5B). Similar as the results for Otx2 expression, overlays of adjacent sections containing mRNA of Aadc and Shh demonstrated that Shh-positive cells in the ventricular zone aligning the third ventricle are in proximity to Aadc positive neurons in the posterior diencephalon (Fig. 5B), whereas Shh expression in the ventricular zone aligning the aqueduct was in proximity of Aadc positive neurons (Fig. 5B, III). The radially orientated TH immunoreactive DA neurons located across the mesencephalic flexure at E12.5 and E14.5 (Fig. 5C) indicate that these neurons migrate radially along the A/P axis (Fig. 5D) and support the idea that few mdDA neurons might be located below the ventricular zone of the third ventricle.

Taken together, these results suggest that mdDA neurons might have different positions during development related to the appearance of Aadc and earlier mapping of Th and Pitx3 in molecularly distinct domains along the anterior/posterior and dorsal ventral axis.

### Spatial Distribution in mdDA Neurons is Linked to Molecular Signature

**Figure 6:**
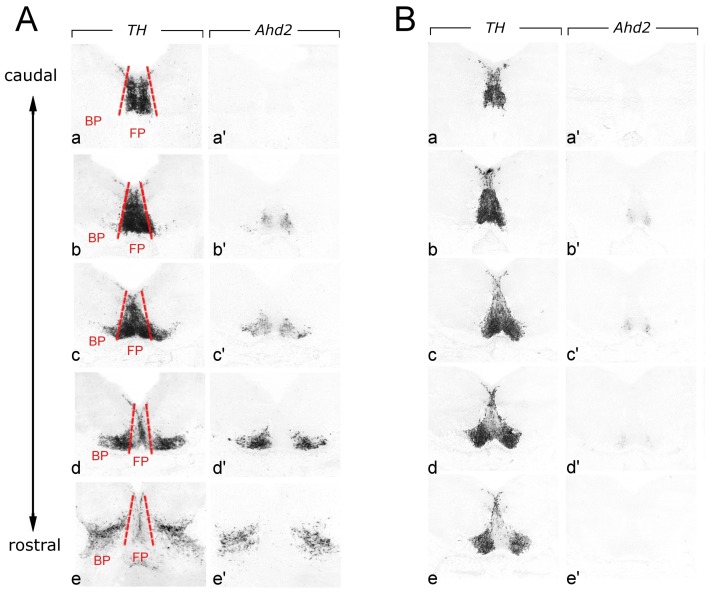
Th and Ahd2 mRNA expression in mdDA neurons of E14.5 wild-type (A) and E14.5 Pitx3-deficient embryos (B) at different levels along the A/P axis. The boundary between floor and basal plate is illustrated by dashed red lines. Abbreviations: BP, basal plate; FP, floor plate.

To determine whether the susceptibility of DA neurons predominantly located in the SNc might be related to their relative position, we studied the mRNA expression pattern of Ahd2. Ahd2 has a restricted expression pattern within the neuronal mdDA population and identifies the DA subpopulation lost in Pitx3-deficient mice and those most vulnerable to degeneration in PD [14]. Figure 6A shows the restricted Ahd2 expression in wild-type mice at E14.5. A clear A/P gradient can be observed in the Ahd2 expression pattern. No Ahd2 expression is observed in the most caudal part of the DA domain (Fig. 6A, panel a’), while Ahd2 expression is gradually increasing in the more rostral parts (Fig. 6A, panels b’–e’). In the most rostral part of the mdDA domain, Ahd2 expression is absent in the medial part, which is rich in Th mRNA expression indicating the presence of mdDA neurons here (Fig6A. panel d’ and e’). As previously shown, the caudal part of the mdDA domain is restricted to the floor plate, while the rostral part of the DA domain consists of floor plate in the medial part of the domain, and basal plate in the more dorsal part (Fig. 3). Taken together, these data show that Ahd2 expression is very scarce in the floor plate (few cells in Fig. 6A panels b’and c’), but very high in mdDA cells positioned in the basal plate. Analysis of Ahd2 expression in E14.5 Pitx3-deficient mice demonstrated that Ahd2 expression was completely lost in the most rostral part of the DA domain (Fig. 6B, panel e’), and nearly absent in the more caudal part (Fig. 6B, panels b’–d’). Interestingly, the few DA cells that did express Ahd2 in Pitx3-deficient mice were restricted to the midline and positioned in the floor plate. As expected, the most caudal part was completely devoid of Ahd2-positive cells (Fig. 6B, panel a’). In conclusion, these findings indicate that the DA cells affected by Pitx3-deficiency are predominantly positioned in the basal plate.

**Figure 7:**
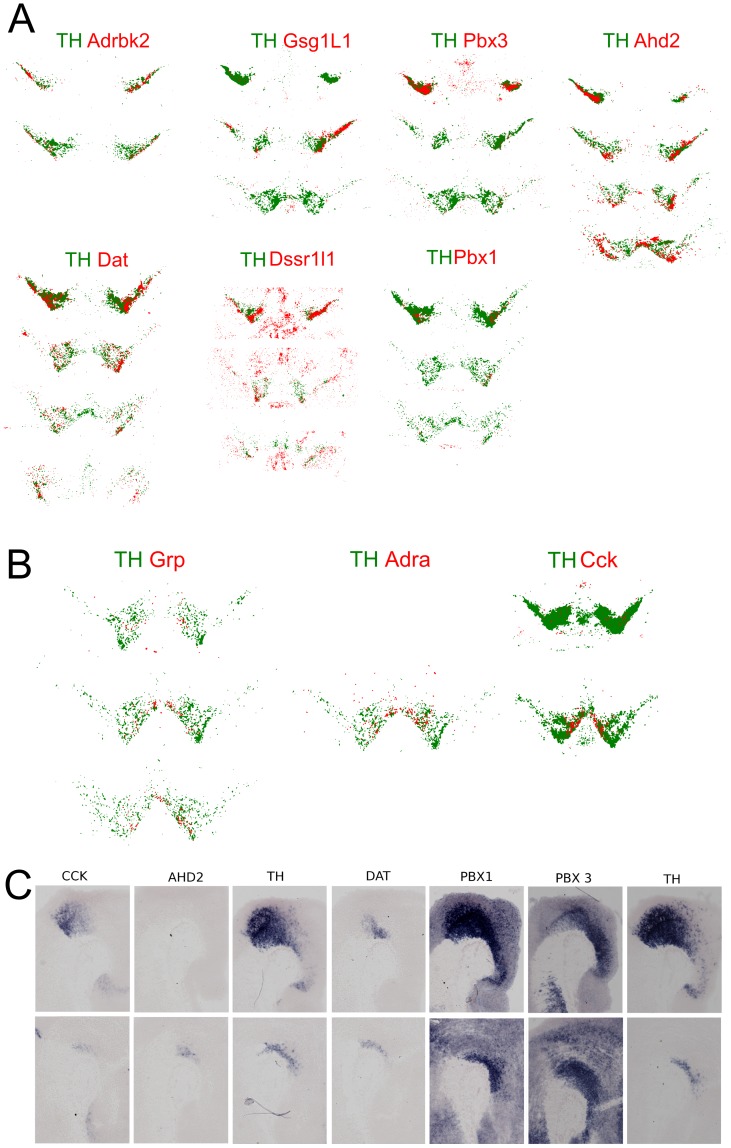
Bitmap traces of expression patterns of adult mdDA subset markers (red) matched to Th (green). (A) Rostral mdDA (SNc) neuronal enriched markers. (B) Caudal mdDA (VTA) enriched markers. (C) E13.5 sagital sections showing Th, Otx2, Cck and Adra expression.

The selective deficits as highlighted by specific effects on SNc populations in the Pitx3 knock-out suggest that developmental location, molecular programming and molecular signature of adult mdDA neurons are linked together. In order to understand the difference in molecular makeup, we aimed to find specific mdDA subsets signatures and relate those to the field of developmental position. Through database searches and analysis of microarray data on SNc/VTA sets we have selected genes for mdDA subset analyzes through in situ hybridization (Fig. 7, Fig. S1-8; Table 1). Bitmap traces of the original ISH data of adjacent section probed for Th and Adrbk2, Gsg21L1, Pbx1/3, Ahd2, Dat and Dssr1L1 indicate that all these markers are clearly enriched in rostral mdDA neuronal groups. The caudal mdDA neuronal population (VTA) has less unique markers and only Grp, Adra1B (Adra) and Cck are clearly marked for their VTA subset specific expression. This suggest that the separate molecular domains lead to the formation of unique molecular signatures among the mdDA neuronal subsets. Some already when mdDA neurons are just specified (Ahd2) and some during late stages of development. In order to investigate the early specification in more detail we performed ISH for Cck, Ahd2, Dat, Pbx1, Pbx3 and Th as control on adjacent E14.5 sagittal sections (Fig7. C). As already mentioned above Ahd2 has the most restrictive expression pattern that is represented to the adult spatial expression pattern. Also the Dat is restricted to the anterior located mdDA neurons at this stage as is represented by the high level of expression of Dat in the adult SNc as compared to the VTA region. Interestingly, also Pbx3 has a clear higher level of expression in lateral/rostral domains which is also represented in the SNc restriction in the adult stage. This is not a general rule since Pbx1 is expressed in the whole mdDA domain and is clearly restricted in the adult stage. Interestingly, Cck,restricted in the adult the caudal mdDA domain, is also restricted to the caudal domain during development. In conclusion, several markers that are restricted to subsets of the mdDA domain are already confined to these regions during development.

**Table 1:** Representation of genes enriched in either the SNc or VTA.

Gene:	SNcenriched:	VTAenriched:	Pitx3regulated:	Nurr1regulated:
Cck	–	+	+	+
Cdh8	+	–	–	–
Dsscr1L1	+	–	–	–
Pbx1	+	–	–	–
Dat	+	+	+	+
Grp	–	+	–	–
Adra1B	–	+	–	–
Hipk2	+	–	–	–
Neuritin	+	–	–	–
Cdh11	+	+	–	–
Adrbk2	+	+	–	–
Pbx3	+	+	–	–
Gsg1L1	+	+	–	–
Slc25a5	+	–	–	–
Homer2	+	–	–	–
Ahd2	+	+	+	+

## Discussion

Young mdDA neurons undergo a multi-stage differentiation process. First, mdDA precursors are generated by proliferative cells in the neuroepithelium. These mdDA precursors then stop dividing, leave the neuroepithelium and become post-mitotic. Next, these post-mitotic young DA neurons differentiate and migrate ventrally to their final position in the midbrain. Finally, late post-mitotic mdDA neurons are specified and adopt their final DA phenotype in the ventral part of the midbrain. During these different phases of DA development, spatio-temporally regulated transcription factors, including Pitx3 specify the developmental fate of these precursors. Why differences in Pitx3-dependence exist between mdDA subsets, besides retinoic acid (RA) production through Ahd2 [7], [14], is still largely unknown, but it is hypothesized here that differences in developmental position may give rise to molecularly distinct mdDA neurons. In the present study, we examined the positions of Aadc, Th, Nurr1 and Pitx3 positive neurons and try to related this to the existence of an adult molecular specification map. We observed mdDA neurons in a continuum across the midbrain, P1, P2 and P3 area, indicating that the rostral mdDA portions develop in the caudal diencephalon, whereas caudally located mdDA neurons develop in the midbrain [19], [31]. Consistent with this, we found in anterior regions Aadc and Th positive neurons near the precursor cells in the neuroepithelium aligning the third ventricle in the diencephalon, whereas these neurons in posterior areas were positioned near the neuroepithelium aligning the aqueduct in the midbrain marked by Th and Aadc. Interestingly, caudally located mdDA neurons (Th and Pitx3 positive) were restricted to the floor plate, whereas more rostral regions contained mdDA neurons in both the floor and basal plate, indicating that mdDA neurons are present, during developmental stages, in regions within the floor and basal plate domains. In addition, the radial pattern of vimentin-immunoreactive fibers from the neuroepithelium to the ventral midbrain [22], suggests that along both the A/P and D/V axis, Aadc and Th positive neurons migrate towards their ventral position, suggested from different neuroepithelial patches. It is thought that neurons forming the VTA and medial SNc are born from the medio-ventral one-third of the neuroepithelium, whereas those forming more lateral parts of the SNc are generated from a more dorsal neuroepithelial patch [36]. We speculate that a molecular neuroepithelial code exists, in which signals expressed in the neuroepithelium are important regulators in providing newly generated mdDA precursors with the appropriate molecular make-up that may give rise to distinct mdDA populations with specific properties.

The differences between the genetic make-up of the brain regions in which the different mdDA subpopulations develop could influence the selective vulnerability to Pitx3-deficiency. Therefore, we studied the mRNA expression pattern of Ahd2. Ahd2 has a restricted expression pattern within the neuronal DA population, and identifies the DA subpopulation lost in Pitx3-deficient mice and those most vulnerable to degeneration in PD [14]. These data allowed us to discriminate between two distinct DA subsets, one which is selectively vulnerable to Pitx3-deficiency (Ahd2-positive), and the other which is not susceptible to Pitx3-deficiency (Ahd2-negative). We found that Ahd2-expressing neurons are predominantly located in the basal plate of the rostral mdDA domain. In the floor plate of the caudal mdDA area only very few Ahd2-positive cells were observed. Interestingly, in Pitx3-deficient mice, Ahd2 expression was completely lost in the basal plate, whereas the expression in the floor plate was unaffected. These data suggest that DA neurons selectively vulnerable to Pitx3-deficiency develop in the basal plate, whereas DA neurons less affected by Pitx3-deficiency are present in the floor plate. These data are in agreement with quantitative analysis of TH-immunoreactive (TH-IR) neurons in Pitx3-deficient mice [14]. In the caudal floor plate area no differences in the number of TH-IR neurons were observed. In contrast, in rostral regions Pitx3-deficient mice displayed a significant loss of TH-IR neurons in the basal plate.

These specific coding differences suggest that specific molecular signatures mark specific subgroups of neurons in the mdDA neuronal population as has already been suggested earlier for Otx2 [37]. We have shown here that many markers exist that are specifically expressed in such subsets in the adult stage as well as during development, although the data described here do not signify the developmental origins of these subsets, the separate positions and the link of restricted gene expression during development and the adult stage do suggest that the molecular signature of mdDA subsets may have their roots in the developmental position along dorsal/ventral and rostral/caudal domains.

## Materials and Methods

### Ethics Statement

All animal studies are performed in accordance with local animal welfare regulations, as this project has been approved by the animal experimental committee (Dier ethische commissie Universitair medisch centrum Utrecht; DEC-UMC-U),and international guidelines.

### Animals

Pregnant C57Bl/6-Jico mice (Charles-River) were euthanized by CO_2_ asphyxiation. Embryos were collected at embryonic day 11.5 (E11.5) to E16.5 (the day on which the copulatory plug was detected was considered embryonic day 0.5).

### In situ Hybridization

Embryos were isolated and immediately frozen on dry ice. Sagittal and coronal sections (16 µm) were cut on a cryostat and collected on SuperFrost Plus slides (Menzel Gläser). In situ hybridization with digoxigenin (DIG)-labeled RNA probes was performed as described previously (Smidt et al., 2004a). Briefly, sections were fixed in 4% paraformaldehyde (PFA) for 10 min and acetylated with 0.25% acetic anhydride in 0.1 M triethanolamine for 10 min. Hybridisation was carried out at 72°C in a hybridization solution containing 50% deionized formamide, 5X SSC, 5X Denhardt’s solution, 250 µg/ml tRNA Baker’s yeast and 500 µg/ml sonificated salmon sperm DNA. Post-hybridization washes were done in 0.2X SSC for 2 hrs at 72°C. DIG was detected with an alkaline phosphatase-labeled antibody (Roche, Mannheim) using NBT/BCIP as a substrate. After DIG in situ hybridization, slides were dehydrated in ethanol, cleared in xylene and mounted using Entellan.

DIG in situ hybridization was performed with the following probes: 222-bp fragment of the rat Th cDNA (Grima et al., 1985), 1350-bp fragment of the rat Pitx3 cDNA (Smidt et al., 2000), Nurr1 fragment containing bp 1022 to the 3′-end of the full-length cDNA (Smidt et al., 2000), Aadc fragment containing bp 22-488 of the mouse coding sequence (Smits et al., 2003), 510-bp mouse Shh fragment containing exon 1 (NM_009170), 930-bp Otx2 fragment of the mouse cds (kindly provided by A. Simeone), 549-bp mouse Dlx2 fragment corresponding to part of exon 1 (NM_010054), Gbx2: bp 777-1199 of the mouse coding sequence (NM_010262), FoxA2 (HNF3-β): bp 380-1201 of the mouse coding sequence (NM_010446). Cck: bp 263-630 of mouse NM_031161, Cdh8: bp 662-1600 of the mouse NM_001039154, Dsscr1l1 bp 247-880 of mouse NM_030598, PBX3: bp 751-1803 of mouse NM_016768, Pbx1: bp1644-2277 of mouse AF020196, GRP: bp 279-758 of mouse BC024515, Adra1b: bp 958-1456 of mouse NM_016991, Hipk2: bp 3102-3540 of mouse NM_001136065, Adrbk2: bp 66-513 of mouse NM_177078, Cdh11: bp 653-1595 of mouse BC057581, Pbx3: bp 751-1803 of mouse NM_016768, Gsg1l1: bp 931-2051 of mouse NM_001101488, Slc25a5: bp 14-560 of mouse NM_007451, Homer2: bp 592-1091of mouse AF093260, Ahd2: bp 568-1392 of mouse NM_013467, Dat: bp762-1127 of rat M80570, Neuritin: bp 637-1250 of mouse NM153529 and Lmx1a, bp 218-1366 of the mouse cDNA NM_033652 (full CDS).

### Nissl Staining

Paraffin-embedded coronal and sagittal E13.5 sections (7 µm) were mounted on SuperFrost plus slides (Menzel Gläser). Sections were deparaffinized, rinsed in water, stained for 10 min in 0.5% cresyl violet and briefly rinsed in an acetate buffer, pH4. The sections were then differentiated in 96% ethanol for 30 s, dehydrated in 100% ethanol, cleared in xylene and mounted with Entellan.

### Immunohistochemistry

Immunohistochemistry with paraffin sections (7 µm) was performed as described earlier (Smidt et al., 2004a). Briefly, sections were deparaffinized through xylene, rehydrated through an ethanol series, washed twice for 5 min in TBS, incubated in 0.3% H_2_O_2_ in TBS for 30 min to reduce endogenous peroxidase activity and washed in demineralized water for 5 min. Next, sections were exposed to microwave treatment in 0.01 M sodium citrate (pH 6) for 3 min at 750W, followed by 9 min at 350W for antigen retrieval. Sections were allowed to cool down to room temperature, washed twice for 5 min in TBS, blocked with 4% fetal calf serum in TBS for 30 min, washed twice for 5 min in TBS, incubated overnight with either polyclonal rabbit anti-TH (1∶1000; PelFreez, Arkansas, USA) or polyclonal rabbit anti-Ki67 (1∶1000; Novocastra Laboratories, UK) in TBS/0.2% Triton. The next day, sections were washed three times with TBS for 5 min, incubated 1 h with biotinylated goat anti-rabbit immunoglobulin (1∶1000), washed three times with TBS for 5 min, incubated for 1 h with avidin-biotin-peroxidase reagents (ABC elite kit, Vector Laboratories, 1∶1000) and washed with TBS three times for 5 min. The slides were processed with a DAB (3,3′- diamino-benzidine) staining procedure until background was lightly stained, washed twice with demineralized water for 5 min, dehydrated with ethanol and mounted using Entellan.

## Supporting Information

Figure S1 **Th and Cck expression pattern of the mdDA region in adult mouse brain adjacent sections.** Sections run from rostral to caudal encompassing the mdDA region.
(TIF)

Figure S2 **Th, Cdh8 and Dsscr1L1 expression pattern of the mdDA region in adult mouse brain adjacent sections.** Sections run from rostral to caudal encompassing the mdDA region.
(TIF)

Figure S3 **Th, Dat and Pbx1 expression pattern of the mdDA region in adult mouse brain adjacent sections.** Sections run from rostral to caudal encompassing the mdDA region.
(TIF)

Figure S4 **Th, Grp and Adra (1B) expression pattern of the mdDA region in adult mouse brain adjacent sections.** Sections run from rostral to caudal encompassing the mdDA region.
(TIF)

Figure S5 **Th and Hipk2 expression pattern of the mdDA region in adult mouse brain adjacent sections.** Sections run from rostral to caudal encompassing the mdDA region.
(TIF)

Figure S6 **Th, Neuritin, Cdh11 and Adrbk2 expression pattern of the mdDA region in adult mouse brain adjacent sections.** Sections run from rostral to caudal encompassing the mdDA region.
(TIF)

Figure S7 **Th, Pbx3 and GsgL1 expression pattern of the mdDA region in adult mouse brain adjacent sections.** Sections run from rostral to caudal encompassing the mdDA region.
(TIF)

Figure S8 **Th, Slc25a4, Homer2 and Ahd2 expression pattern of the mdDA region in adult mouse brain adjacent sections.** Sections run from rostral to caudal encompassing the mdDA region.
(TIF)
