# Supplementary figures and images for: Molecular Marker Differences Relate to Developmental Position and Subsets of Mesodiencephalic Dopaminergic Neurons
Source: PLoS One. 2013 Oct 7;8(10):e76037. doi: 10.1371/journal.pone.0076037 (PMC3792114; doi:10.1371/journal.pone.0076037)

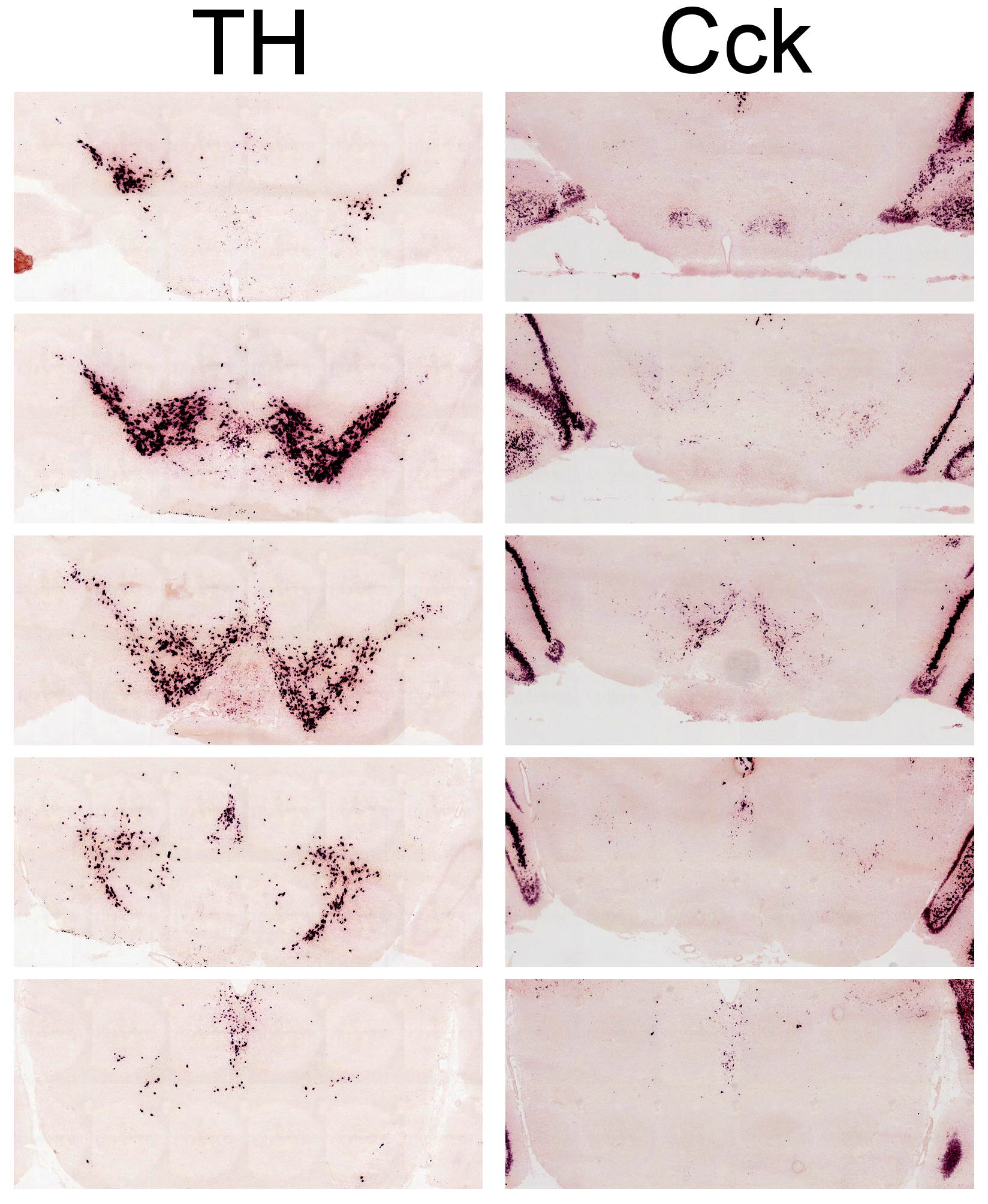

Supplement: Figure S1 — Th and Cck expression pattern of the mdDA region in adult mouse brain adjacent sections. Sections run from rostral to caudal encompassing the mdDA region. (TIF) [file pone.0076037.s001.tif]

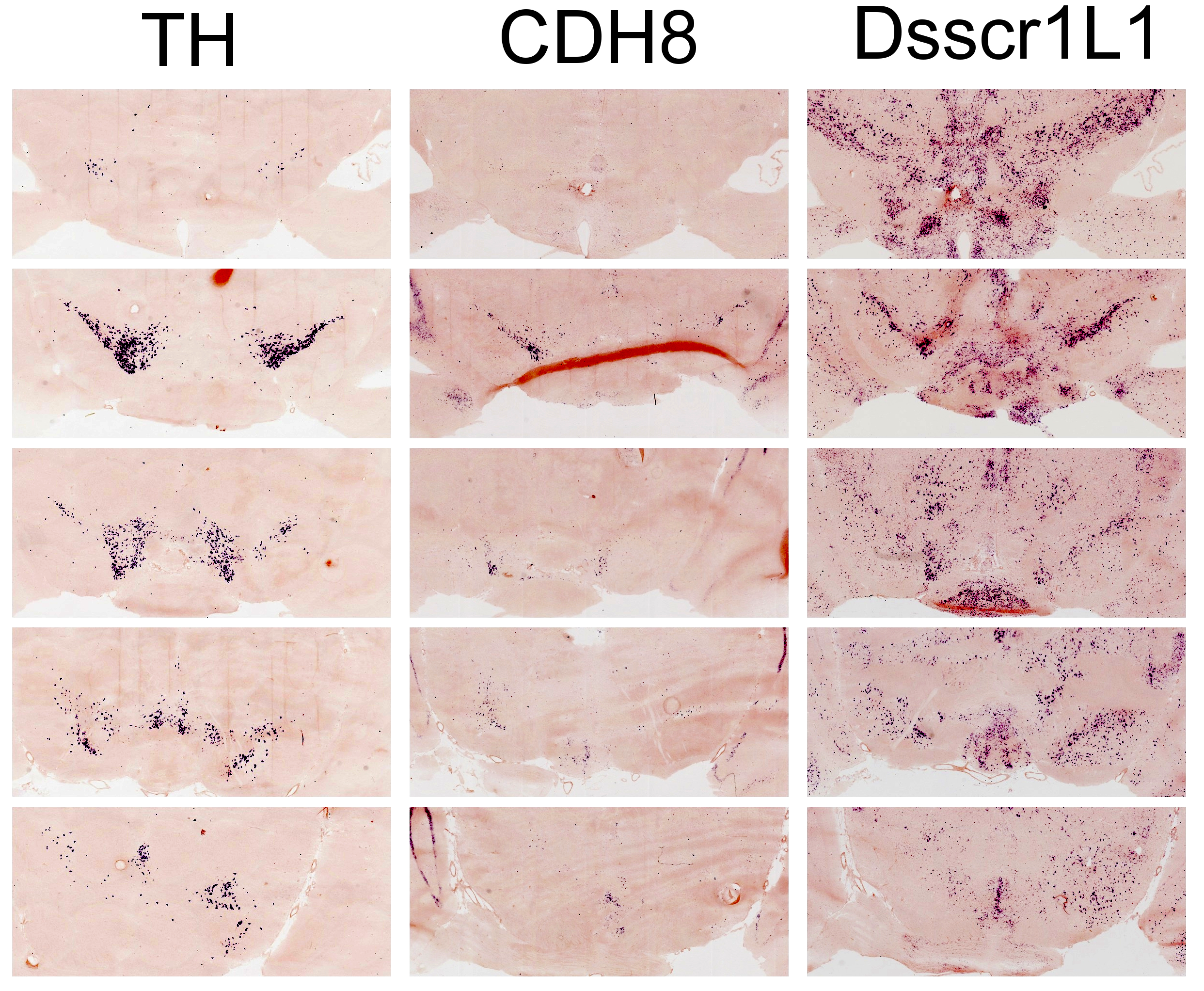

Supplement: Figure S2 — Th, Cdh8 and Dsscr1L1 expression pattern of the mdDA region in adult mouse brain adjacent sections. Sections run from rostral to caudal encompassing the mdDA region. (TIF) [file pone.0076037.s002.tif]

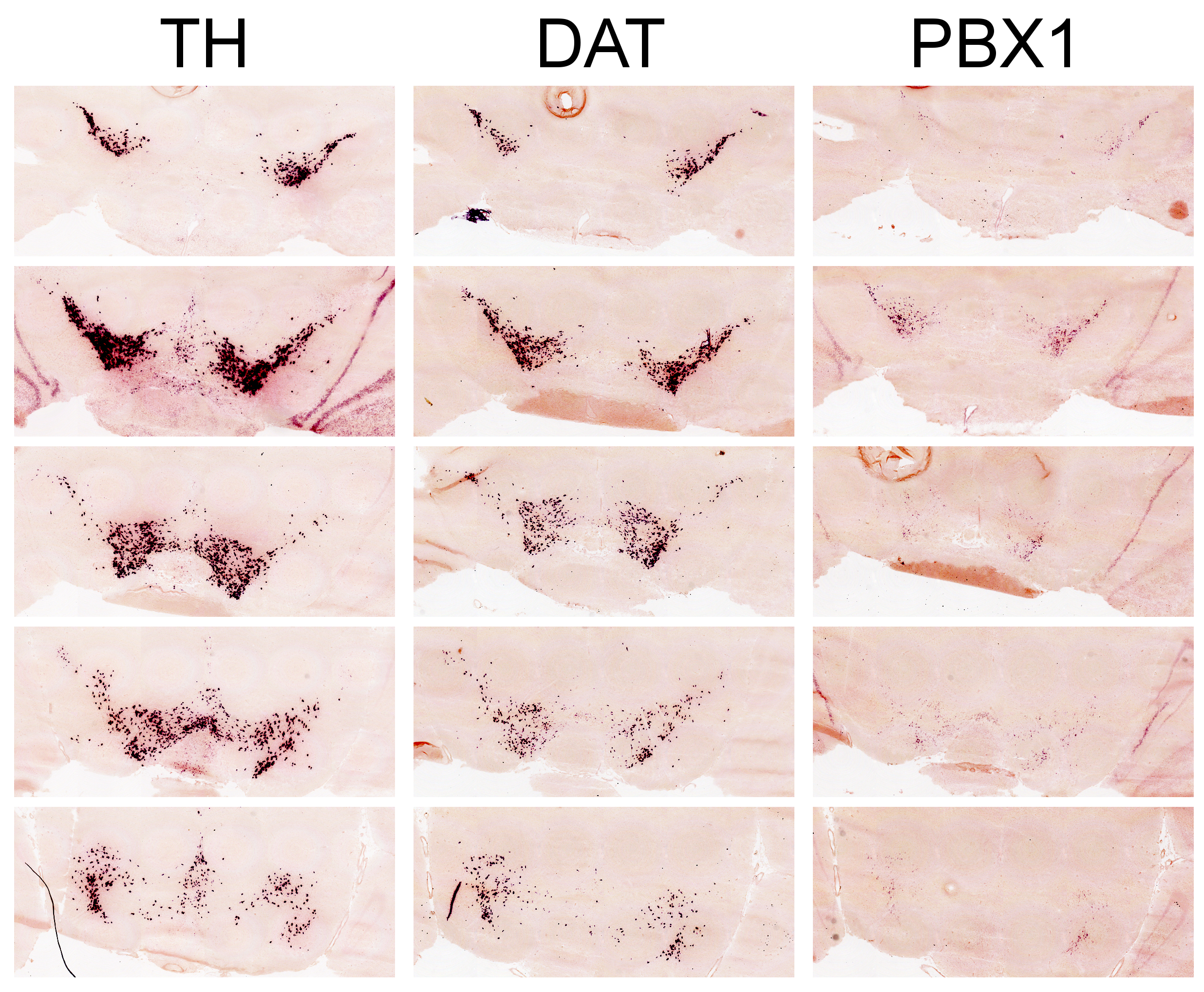

Supplement: Figure S3 — Th, Dat and Pbx1 expression pattern of the mdDA region in adult mouse brain adjacent sections. Sections run from rostral to caudal encompassing the mdDA region. (TIF) [file pone.0076037.s003.tif]

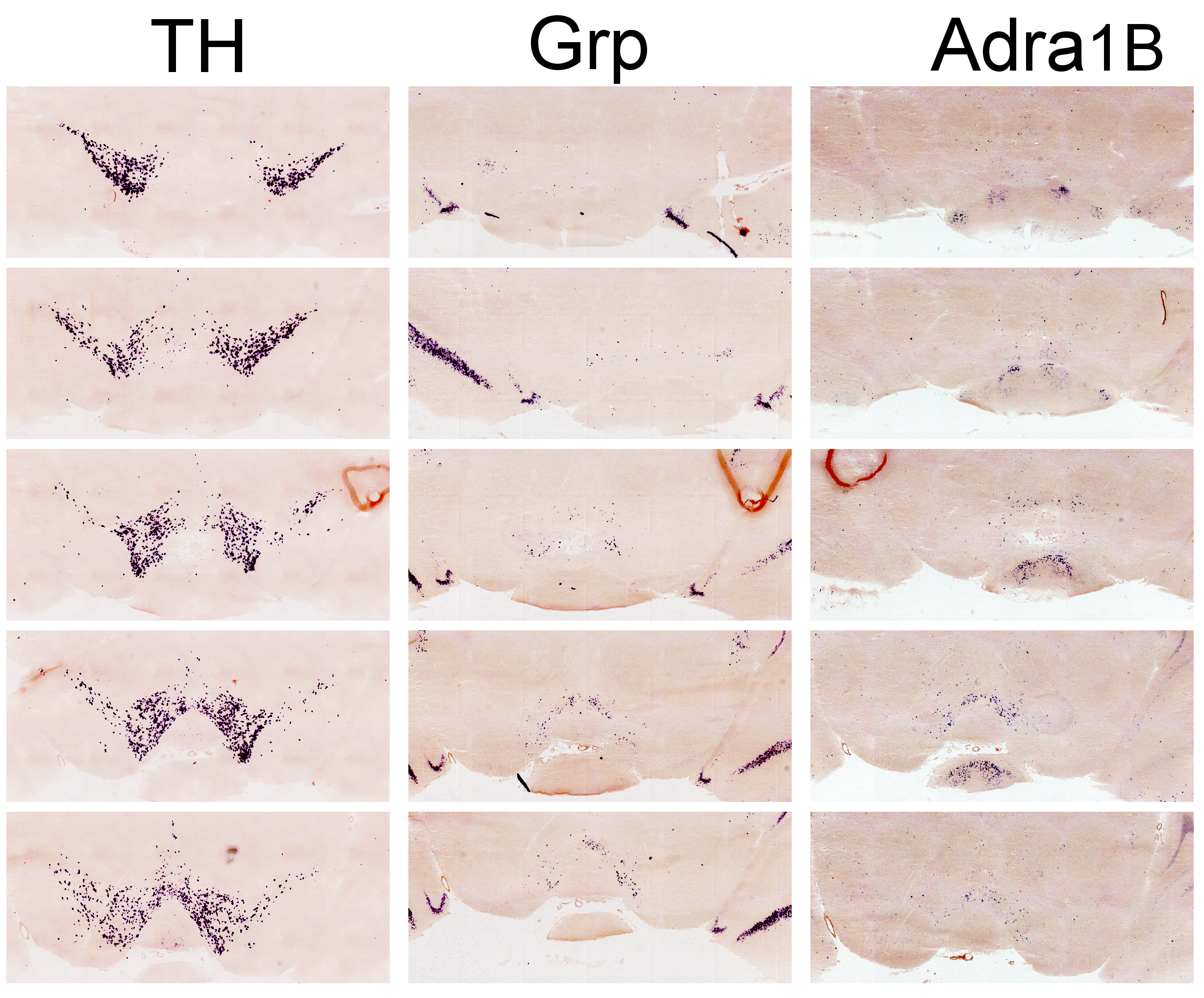

Supplement: Figure S4 — Th, Grp and Adra (1B) expression pattern of the mdDA region in adult mouse brain adjacent sections. Sections run from rostral to caudal encompassing the mdDA region. (TIF) [file pone.0076037.s004.tif]

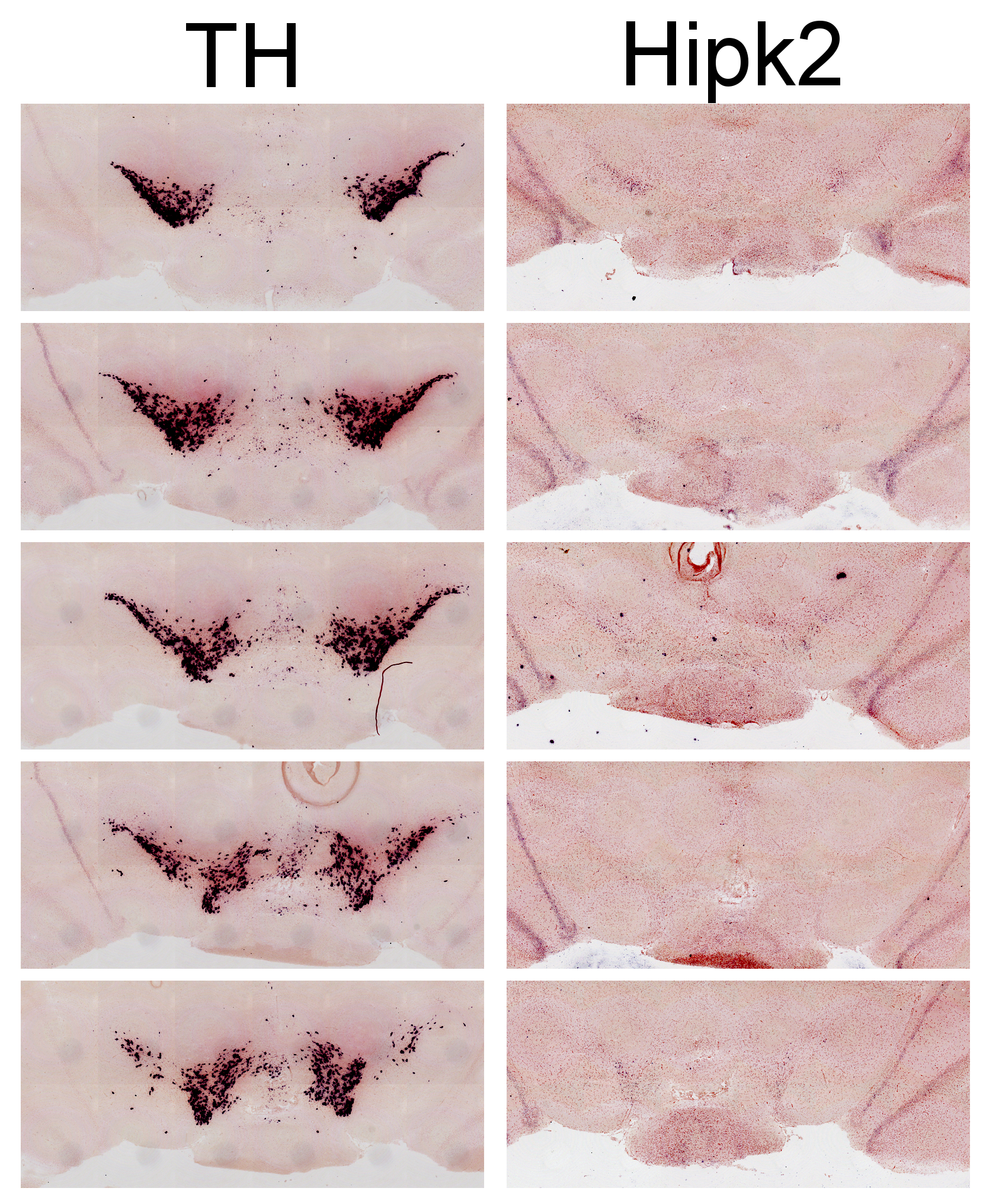

Supplement: Figure S5 — Th and Hipk2 expression pattern of the mdDA region in adult mouse brain adjacent sections. Sections run from rostral to caudal encompassing the mdDA region. (TIF) [file pone.0076037.s005.tif]

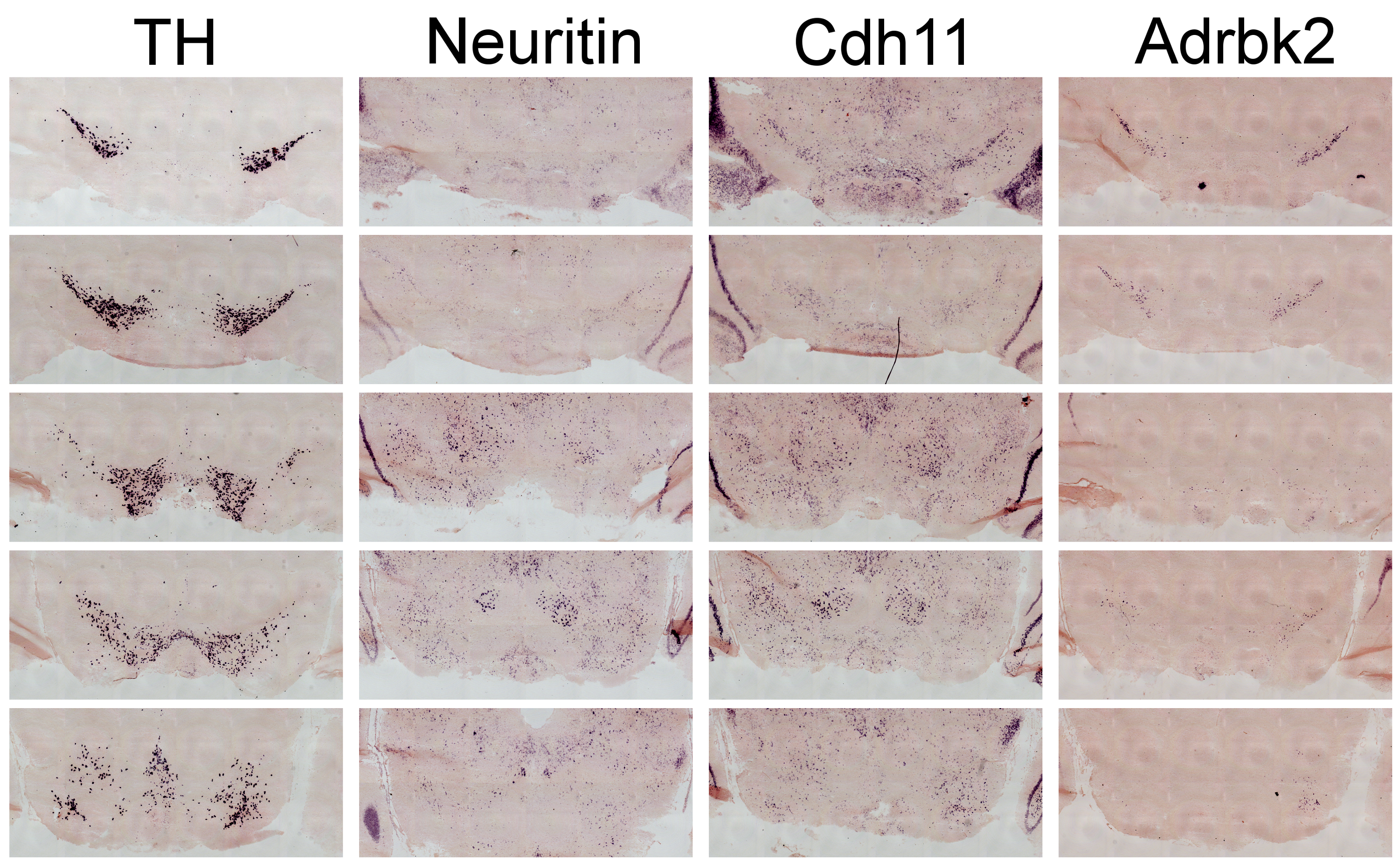

Supplement: Figure S6 — Th, Neuritin, Cdh11 and Adrbk2 expression pattern of the mdDA region in adult mouse brain adjacent sections. Sections run from rostral to caudal encompassing the mdDA region. (TIF) [file pone.0076037.s006.tif]

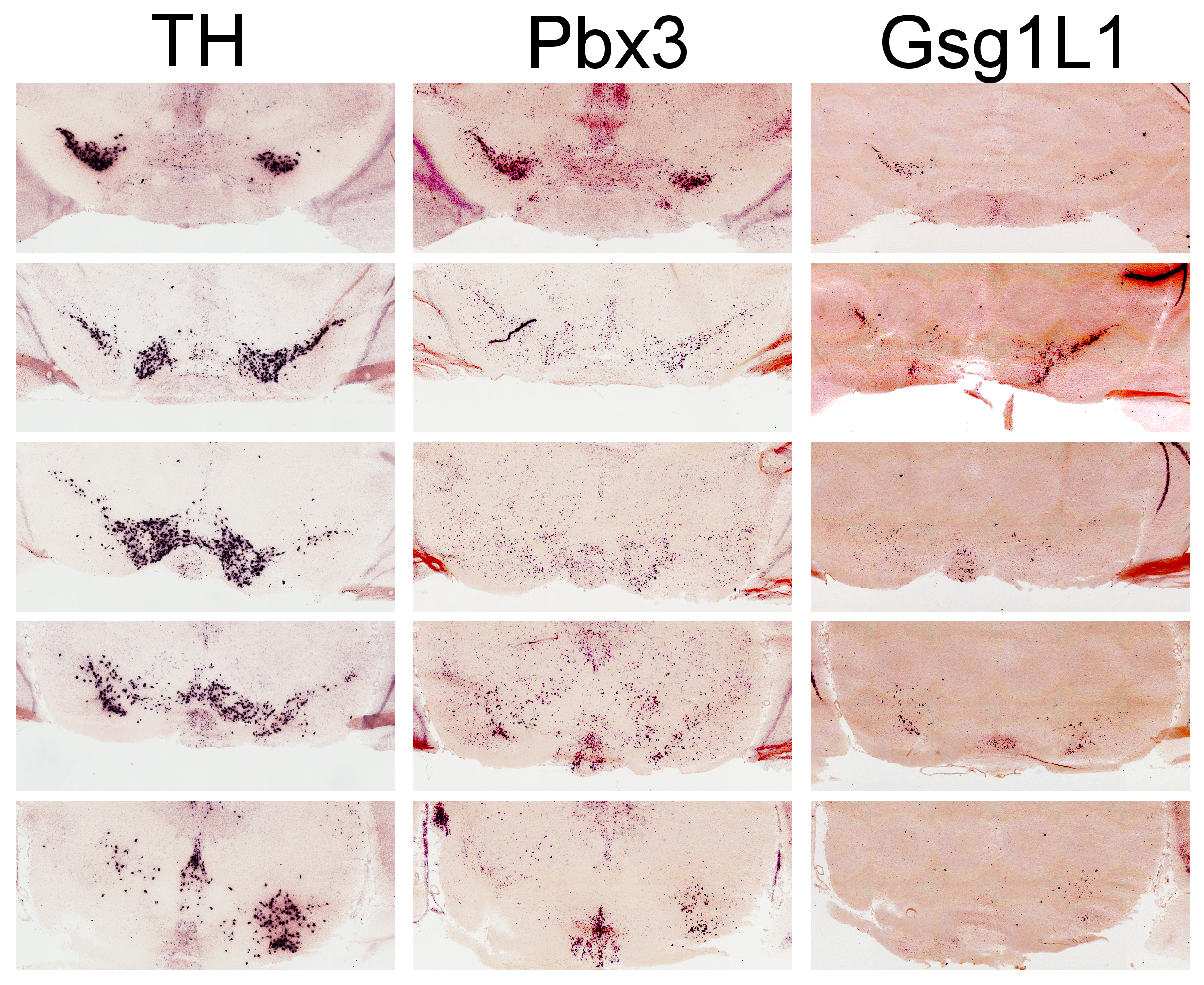

Supplement: Figure S7 — Th, Pbx3 and GsgL1 expression pattern of the mdDA region in adult mouse brain adjacent sections. Sections run from rostral to caudal encompassing the mdDA region. (TIF) [file pone.0076037.s007.tif]

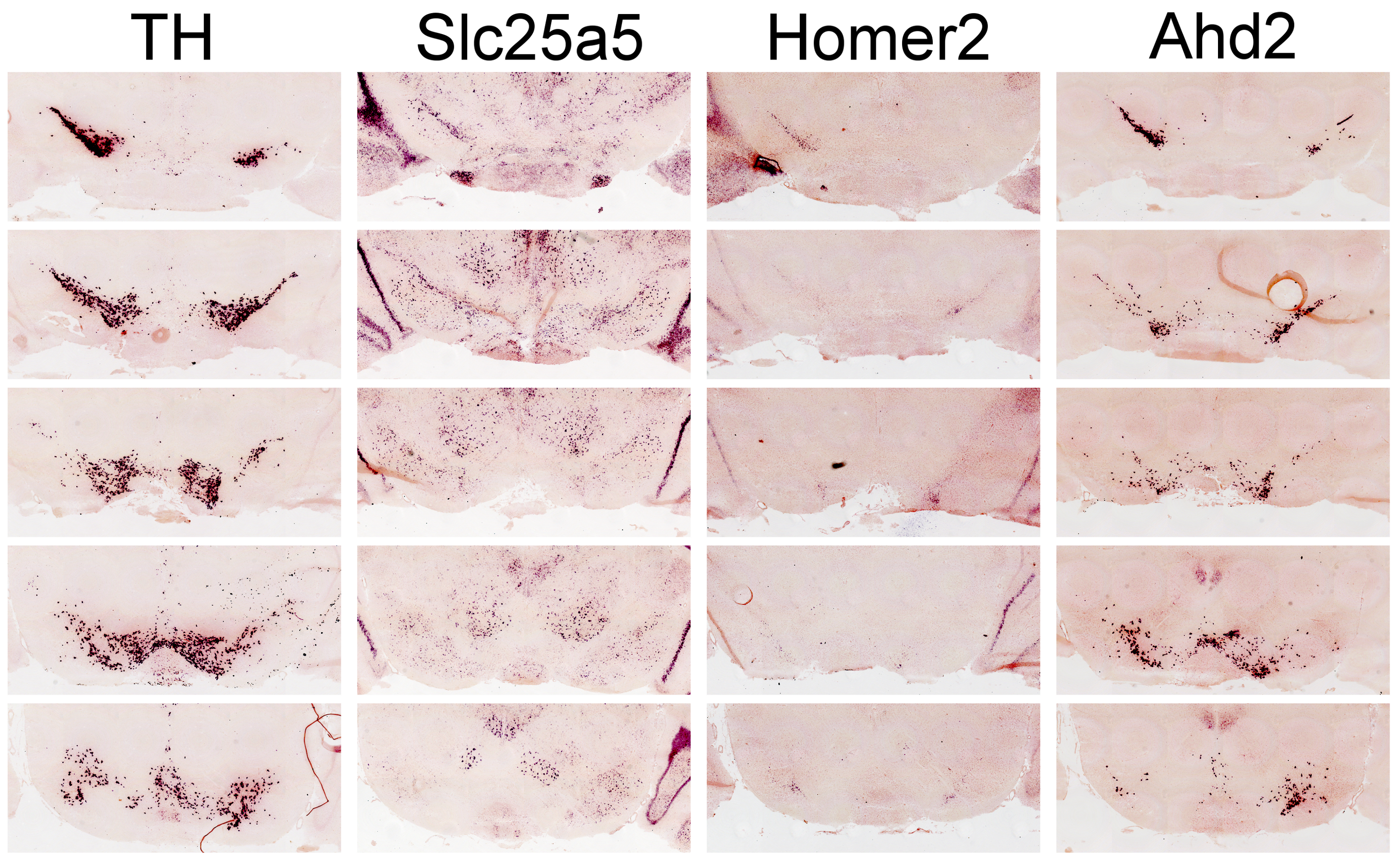

Supplement: Figure S8 — Th, Slc25a4, Homer2 and Ahd2 expression pattern of the mdDA region in adult mouse brain adjacent sections. Sections run from rostral to caudal encompassing the mdDA region. (TIF) [file pone.0076037.s008.tif]
